# Study of backspatter using high-speed video of experimental gunshots

**DOI:** 10.1007/s12024-020-00326-0

**Published:** 2020-12-14

**Authors:** Christian Schyma, Fabienne Baumann, Burkhard Madea, Walther Gotsmy

**Affiliations:** 1 Institute of Forensic Medicine, University of Bern, Bühlstrasse 20, 3012 Bern, Switzerland; 2 Institute of Legal Medicine, University of Bonn, Stiftsplatz 12, 53111 Bonn, Germany; 3 Department of Forensic Medicine and Forensic Neuropsychiatry, Paris-Lodron University of Salzburg, Ignaz-Harrer-Straße 79, 5020 Salzburg, Austria; 4 Institute of Forensic Medicine, University of Bern, Bühlstrasse 20, CH-3012 Bern, Switzerland

**Keywords:** Suicide, Firearm, Backspatter, Biological traces, Muzzle gases, Wound ballistics

## Abstract

Backspatter is biological material that is ejected from the entry wound against the line of fire. This phenomenon was also observed in wound ballistic simulations using so called "reference cubes" (12 cm edge length, 10% gelatin, 4 °C, paint pad beneath the cover). High-speed video records from 102 experimental shots to these target models using full metal jacketed bullets in the calibers .32 auto, .38 special, 9 mm Luger and .357 Magnum were analyzed for chronology, morphological appearance and velocity of fluid ejection. Generally, a short tail splashing of surface material occurred when the bullet was penetrating the target. In 51 shots from distance (≥ 5 cm), regardless of caliber and shot range, a linear jet of fluid started in connection with the first collapse of the temporary cavity. The initial velocity of the jet was measured between 6 and 45 m/s. The jet was streaming on for about 60 to 100 ms with a stochastic deviation of ± 13° to the horizontal. Close range and contact shots showed earlier and faster (up to 330 m/s) backspatter depending on the cartridge and the gap between muzzle and target. Gaseous aerosol-like spray and cone-like spatter indicated an increasing influence of muzzle gases with decreasing shot range. Even under standardized experimental conditions, variations of backspatter were observed in near/contact shots.

**Electronic supplementary material:**

The online version of this article (10.1007/s12024-020-00326-0) contains supplementary material, which is available to authorized users.

## Introduction

Wound ballistics investigates the interaction of projectile and tissue. The injuring capacity of a bullet arises from its kinetic energy. If the energy density of a bullet is larger than the tissue specific value, the bullet penetrates [1]. Such a non-elastic impact of a bullet on a biological target leads to the tissue being crushed [2, 3]. Crushed tissue is then dispersed within the wound channel [4]. In real gunshot injuries the penetration of a bullet causes multiple vessel lacerations with subsequent bleeding, primarily inside the wounded area [2], together with external bleeding at the entry and exit wounds. In summary, a perforating projectile can cause crushed tissue and bloody fluid to accelerate into the direction of fire (“forward spatter” [5, 6]) or against the direction of fire (“backspatter” [7, 8]). As expected, forward spatter is associated with the exit wound and backspatter with the entry wound. Backspatter can be observed in many (but not all) cases of gunshots to the head [7, 8].

During the experimental investigation of staining inside firearm barrels [9], transparent target models (“reference cube” [10]) were introduced. The reference cube was equipped with a thin foil bag containing acrylic paint, barium sulfate and blood, which was mounted beneath the front cover made by an absorbent kitchen wipe. Although the reference cube was originally designed to create optically visible traces in gun barrels, it was also possible to observe the propagation of colored material in and against the direction of fire. At the same time, high-speed video (HSV) showed the passage of the projectile and the formation of the temporary cavity (TC). HSV enables measurements of the bullet velocity [11] and its deceleration [12]. Previous studies illustrated the additional influence of muzzle gases on the TC in contact [13] or near contact shots [14]. The observed properties of the target model can contribute to a more comprehensive understanding of “backspatter”.

The present study investigates back spattered fluid in the context of experimental gunshots to the 12 x 12x12 cm^3^ gelatin reference cube.

## Material and methods

In standardized conditions (4 °C), "reference cubes" of 10% gelatin [10] doped with a liquid mixture of acrylic paint were shot from various distances, using calibers .32 auto (7.65 mm Browning), .38 special, 9 mm Luger (9 × 19) and .357 Magnum handguns. Only non-deforming bullets were used. The shooting experiments were recorded as sequences of uncompressed 12-bit-TIFF-images using two Fastcam SA-X2 (Photron Europe Ltd., West Wycombe, UK) set to 40 000 frames per second (fps) and 10 µs exposure time; one camera was positioned orthogonally to the line of fire, the other was pointing to the muzzle of the gun or the entry in the target. Videos were examined with Photron Fastcam Viewer PFV Version 3. Via the "snapshot" feature single frames were exported to 8-bit-TIF-format and analyzed using AxioVision 64SE rel. 4.9 (Carl Zeiss, Oberkochen, Germany) as previously published [12].

## Results

In total 102 high-speed video records (principal camera, 465 GB, 320 000 frames) were systematically analyzed. This required repetitive examination (dozens of times) by two independent investigators over two years. The description and identification of relevant events were consistent and reproducible within a variation of ± two frames (± 50 µs). Videos recorded by the second camera were only inspected in cases of doubt.

### Chronology of distinct phenomena and their appearance

**Fig. 1:**
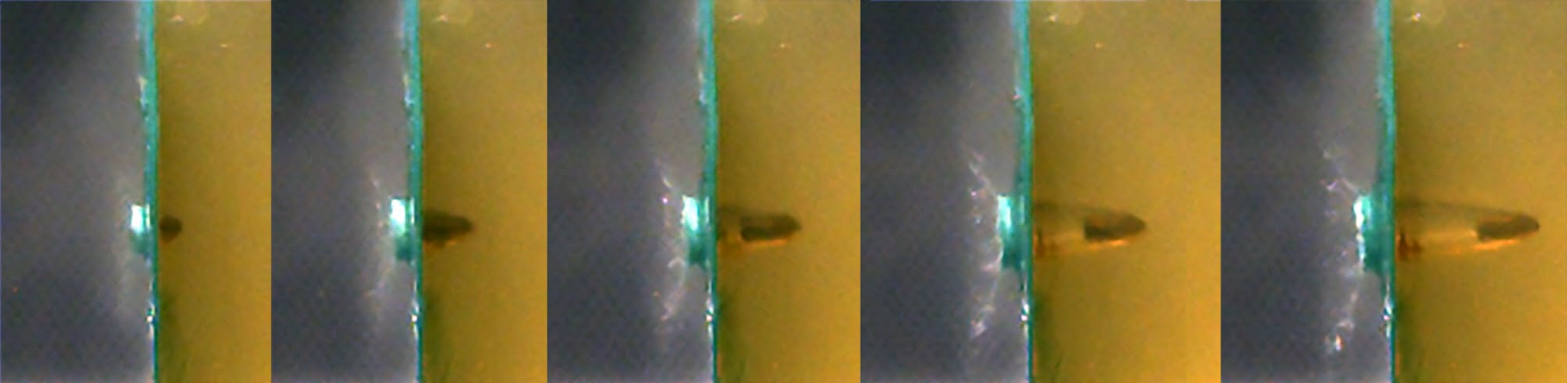
32 auto full metal jacketed bullet penetrating into the target model (covered by a green kitchen wipe) and causing tail splashing. 25 µs between each frame

**Fig. 2:**
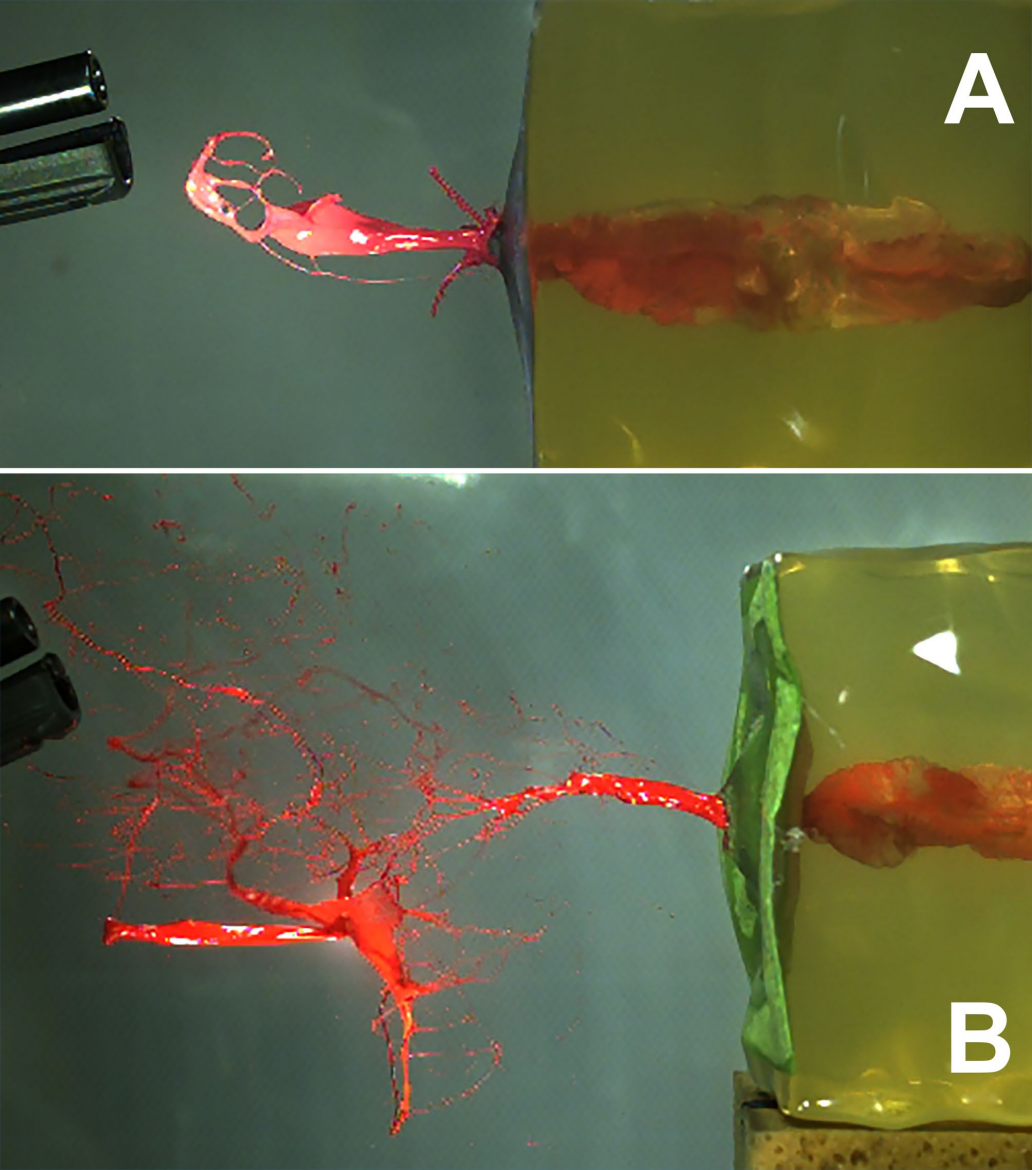
A: Wide linear jet (7 m/s) with curved end. 9 mm Luger, 5 cm distance, 16.7 ms. B: Linear jet dissociating in a web of liquid filaments. 9 mm Luger, 10 cm distance, 26.2 ms. Maximum velocity 19.8 m/s (measured at 12 ms) decreased to 7.1 m/s (picture)

First, a point zero in time has to be defined. Whereas it is obvious for shots from distance that this point is the moment when the bullet penetrates into the target model, the situation is different for contact or near contact shots. Muzzle gases act on the target before the bullet penetrates. Hence, the frame before the first detectable change inside the target was set to zero. The determination of the moment when the bullet entered and exited the gelatin block did not provide additional information. The entry of the bullet caused a crown-like eversion of the surface-coat against the direction of fire with short radial spattering of surface material (kitchen wipe and gelatin) (Fig. 1, Online Resource 1 and 2). Backspatter of fluid was not observed in any of the experimental shots in the phase of bullet penetration. Ejected material against the line of fire was noted: jet (linear), gas, gaseous fluid (aerosol-like), spray or spatter. The second camera in the oblique position showed that the aerosol and spray had a cone shaped spatial extension, whereas the jets travelled along a line (Fig. 2A). The deviation of the jet was measured with respect to the horizontal line (0°). In the shots, in which a jet was found, all calibers provoked either descending (n=37, median -13°) or ascending jets (n=37, median +12°). Extreme Angles (maximum ± 45°) were rare. Four jets were practically horizontal. In four cases, three times with .357 Magnum and once with 9 mm Luger, the jet had a V-form with a descending and an ascending branch. However, considering the jet as only a one-dimensional stream would be too simplistic. The end of the jet travelling towards the firearm was often curved and sometimes broke into a slowly moving web of filigree liquid filaments (Fig. 2B). Furthermore, the jets were of varying width and often showed a slow rotation around their longitudinal axis (Online Resource 3).

**Fig. 3:**
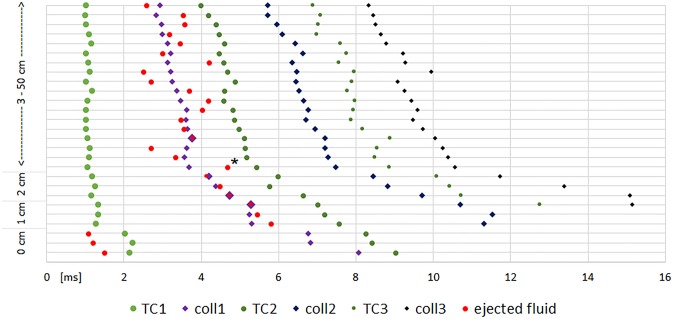
Timeline of 27 shots in the caliber .38 special. The y-axis displays the shot range. TC = maximum of the temporary cavity. Coll = collapse of the TC. * value corrected by oblique camera

**Fig. 4:**
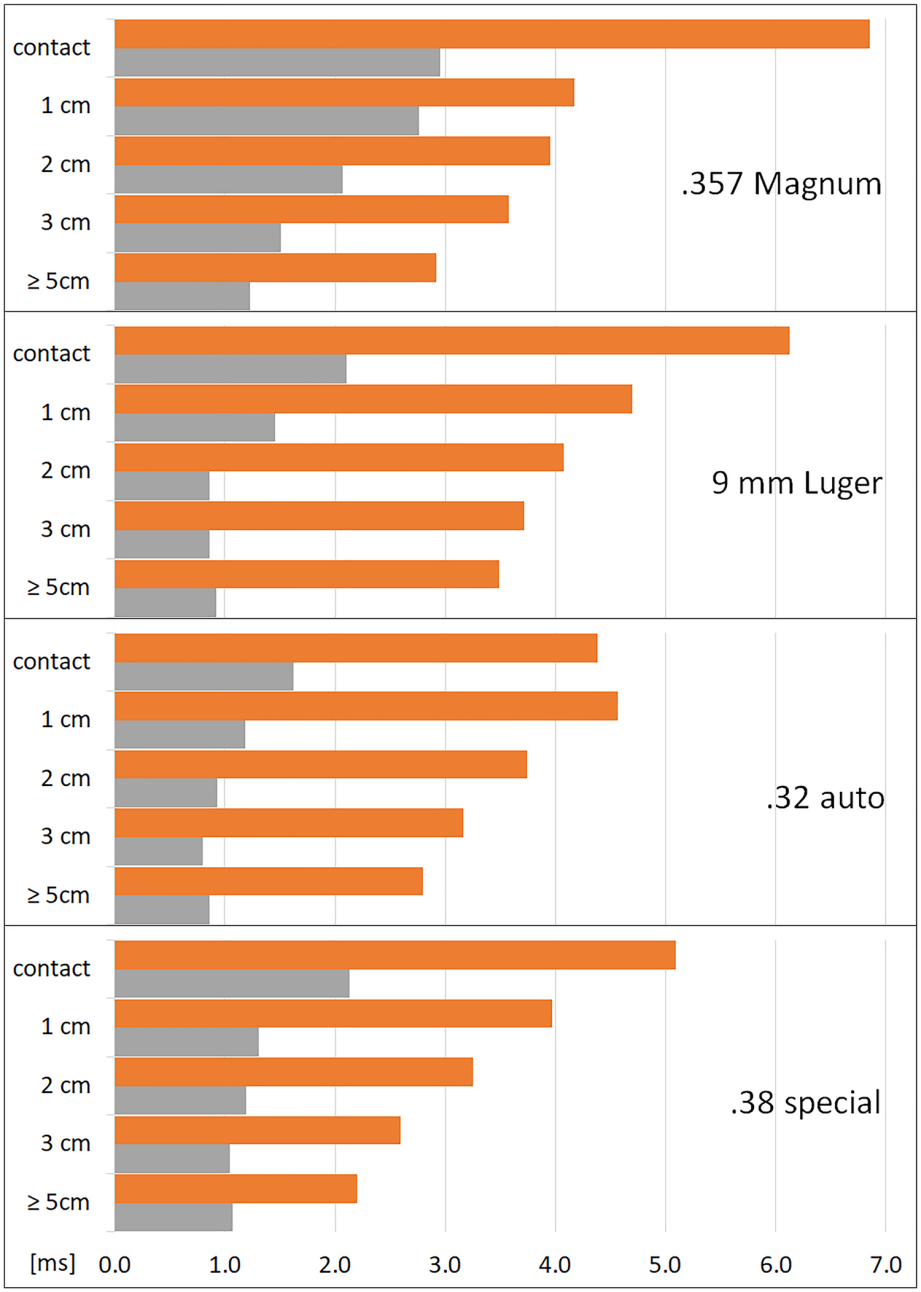
Time until first full expansion of the TC in longitudinal direction (grey) and time needed for first collapse (orange). X-axis displays the time (mean), Y-axis the shot range

The moment of the maximal longitudinal expansion of the temporary cavity (TC) and the moment of its first collapse were noted. The subsequent oscillations of the TC showed discernible maxima and minima only for shot ranges of 5 cm and above. Fig. 3 displays the time line of .38 special shots with obvious differences for close range shooting as an example. Fig. 3 gave the impression that the longer time the TC needs for full expansion the longer the collapse takes. This can be verified in Fig. 4, which shows the duration to reach the first maximum and the first collapse. The time for maximum TC is significantly longer in contact shots, independent of the caliber. Furthermore, TCs produced by .357 Magnum shots from 1 or 2 cm distance and by a 9 mm Luger from 1 cm distance take longer to develop than in shot ranges beyond 5 cm. Surprisingly, the influence of the decreasing muzzle-to-target-distance on the increasing time for the complete first collapse of the TC is clearly visible for all calibers.

**Fig. 5:**
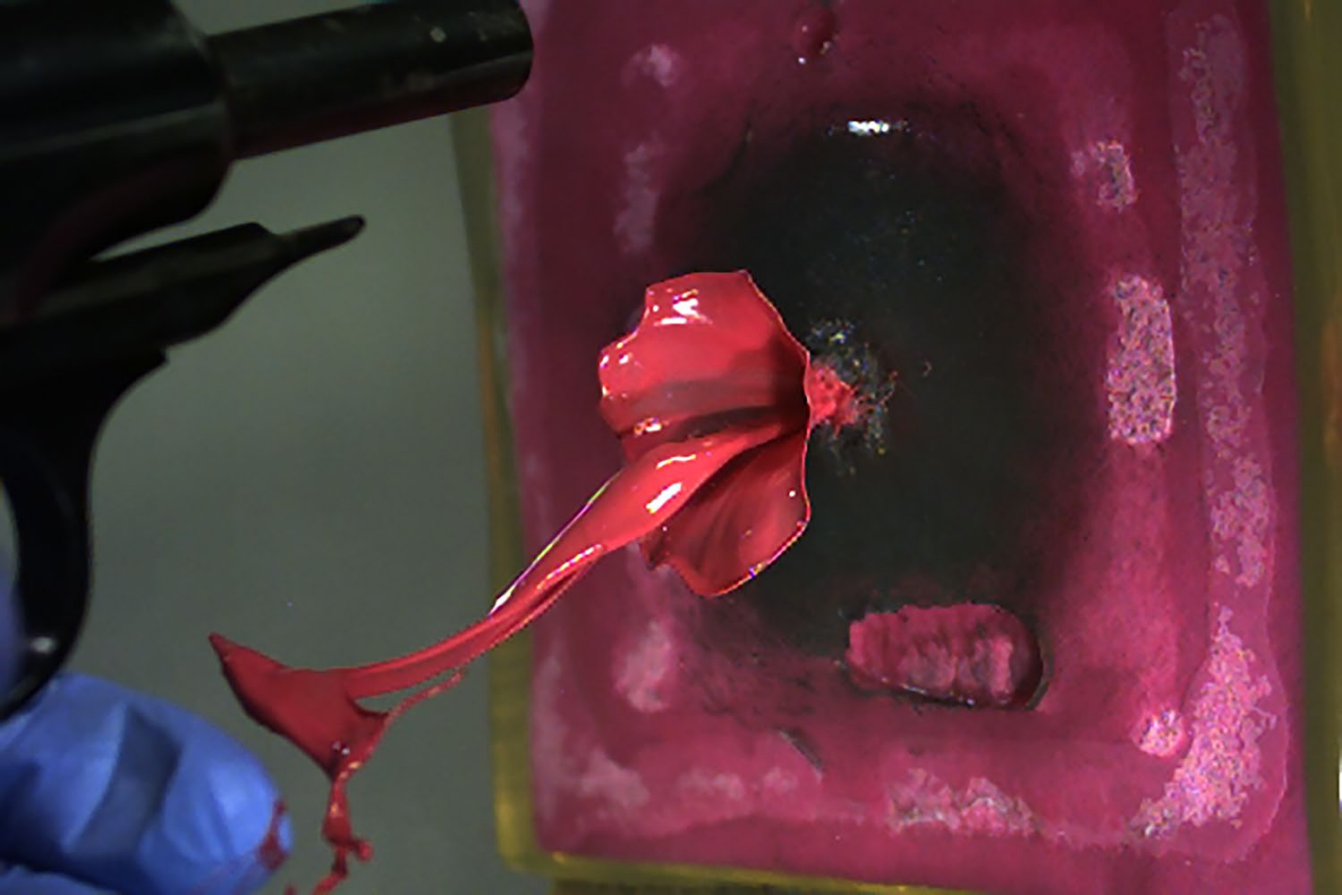
Tulip-like widening of a jet 15.7 ms after close range shot using .32 auto

**Fig. 6:**
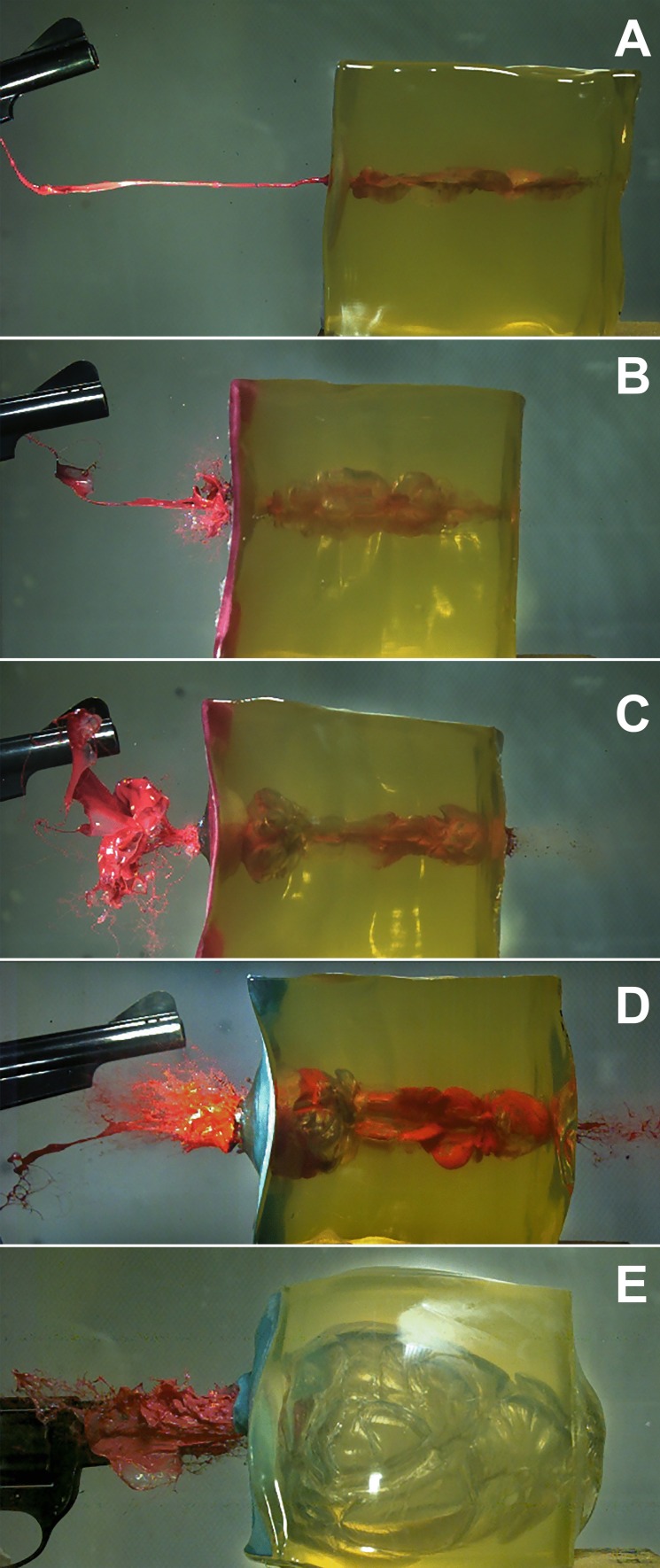
Increasing, but variable influence of muzzle gases in the example of .38 special: A: 5 cm distance, 26.4 ms, fine horizontal jet, 6.1 m/s. B: 2 cm distance, 13.1 ms, jet plus fine spray, 31.5 m/s. C: 1 cm distance, 13.7 ms, wide jet transforming into spray, 26.8 m/s. D: 1 cm distance, 12.6 ms, fast spray (45.4 m/s) following the jet. E: contact, 3.4 ms, abundant spatter, 48.2 m/s

In the caliber .38 special (four inch barreled revolver), the retrograde ejection of material starts mostly after the complete collapse of the TC without discernible variation in different shot ranges ≥ 1 cm. The marked shot in Fig. 3 (10 cm distance) provoked a very late exit of fluid after the second maximum of TC (orthogonal observation). The oblique camera view revealed the start of the slow jet 0.75 ms before the second maximum. In some cases the ejection of fluid is preceded by a fog-like exhaustion. With the exception of the contact shots, the fluid is primarily pressed out as a linear jet, which can travel horizontally, or in a descending line for more than half a meter. At a shot distance of 3 cm a small gaseous widening of the jet was noted at 26.9 ms (22 ms after the jet's start). For shots with 1 and 2 cm distance, spraying was observed at about 12 ms (about 7 ms after the jet's start). Mostly, a ring- or tulip-like shape (Fig. 5) blow up of the jet was observed. Contact shots regularly produced a wide spray cone early (1.25 ms), shortly after the projectile’s exit during the expansion of the TC. Fig. 6 demonstrates the diversity of findings.

The results from caliber .32 auto shots (7.65 mm Browning, Walther PP pistol) were similar to those of .38 special with exception of 1 cm distance shots where spraying comparable to contact shots was observed (Online Resource 4). Initially a very fine spray, combined with a lot of gas occurred very early (0.6 ms), followed by large amounts of liquid late after the collapse of the TC. This chronologic phenomena was also observed with .32 auto contact shots. The moment of the intensive spattering after contact shots (7.2 ms) was associated with the TC's collapse, markedly later than with .38 special.

**Fig. 7:**
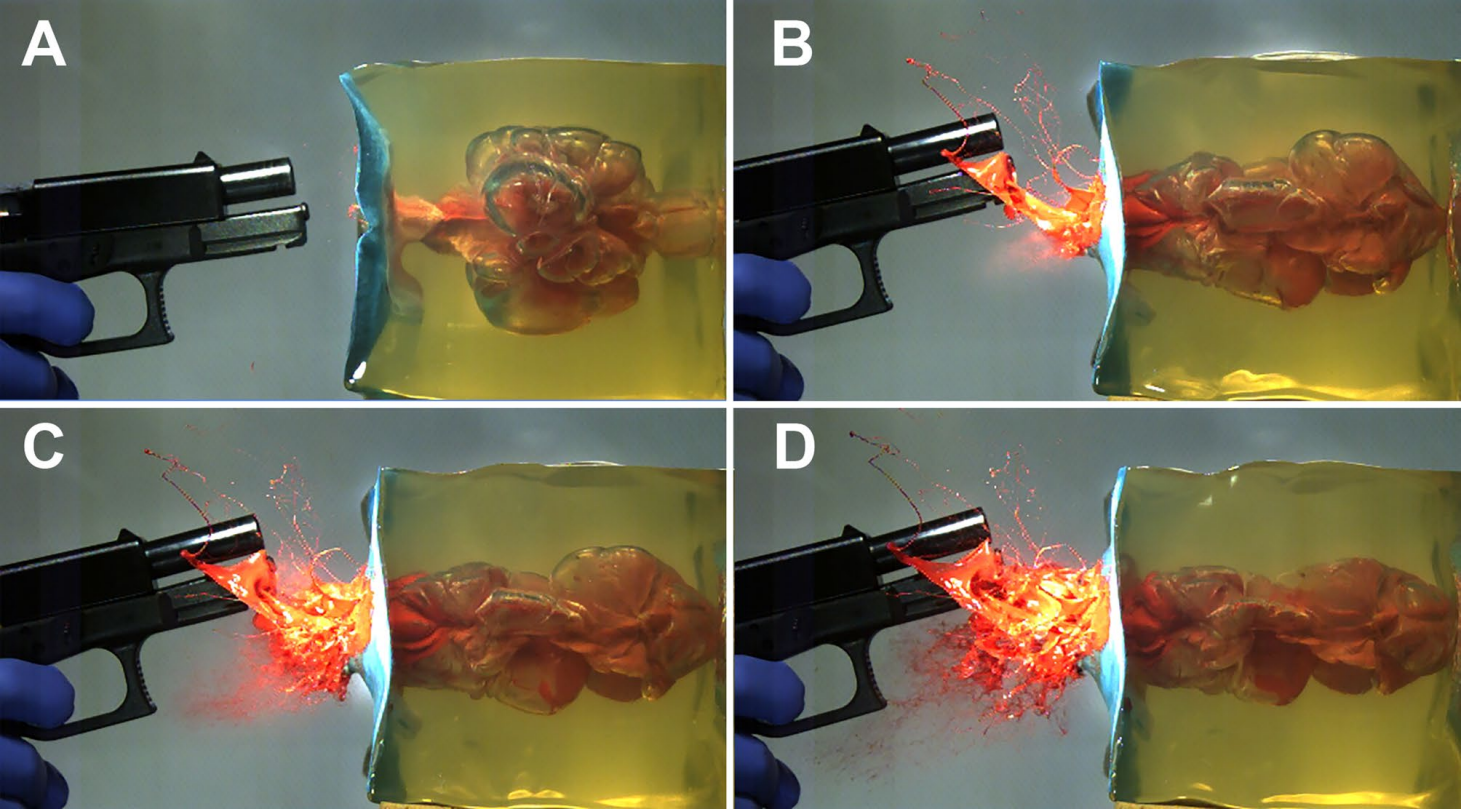
9 mm Luger shot with 1 cm distance. After the collapse of the TC (6 ms) the TC is expands again (A 7.2 ms), a jet is squeezed out and is transformed (B 12.5 ms, C 13.3 ms, D 14.2 ms) into spray by escaping gases

**Fig. 8:**
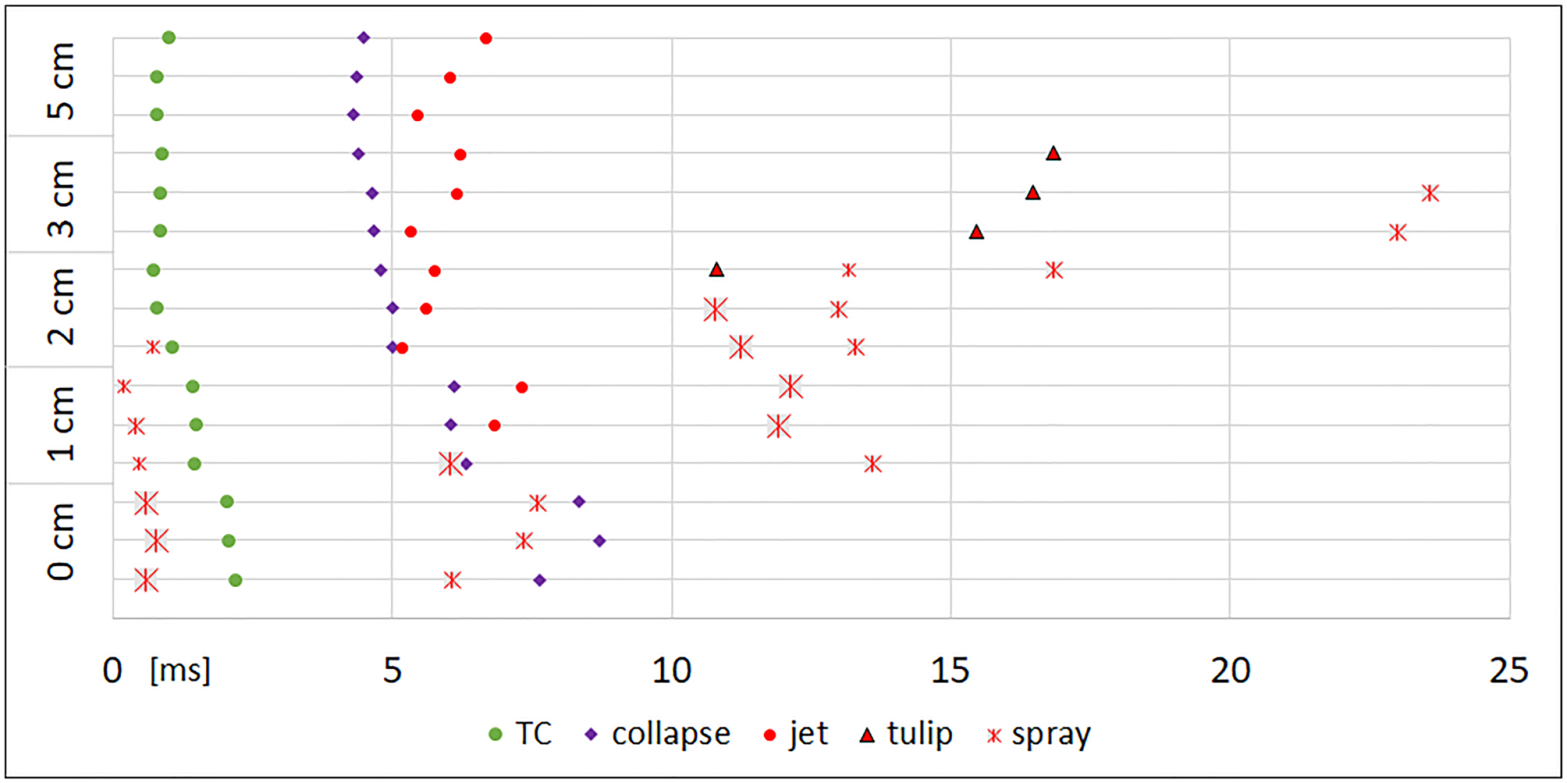
Timeline of the phases of fluid ejection in shots using the caliber 9 mm Luger

Shots from a 9 mm Luger (Glock 17 pistol), from a distance ≥ 5 cm, provoked linear jets a short time before or after the collapse of the TC. In some cases, an aerosol of fine paint drops preceded the jet independent of the shot range. Shots from 3 and 2 cm caused a jet, which transformed into spray. With 9 mm Luger shots from 1 cm distance, spraying began in the early expansion phase of the TC, followed by larger spray effects after the TC's collapse (Fig. 7). Contact shots showed an early (0.65 ms) wide and abundant spray cone. Figure 8 shows the earlier start of spraying with a decrease of the muzzle to target distance.

**Fig. 9:**
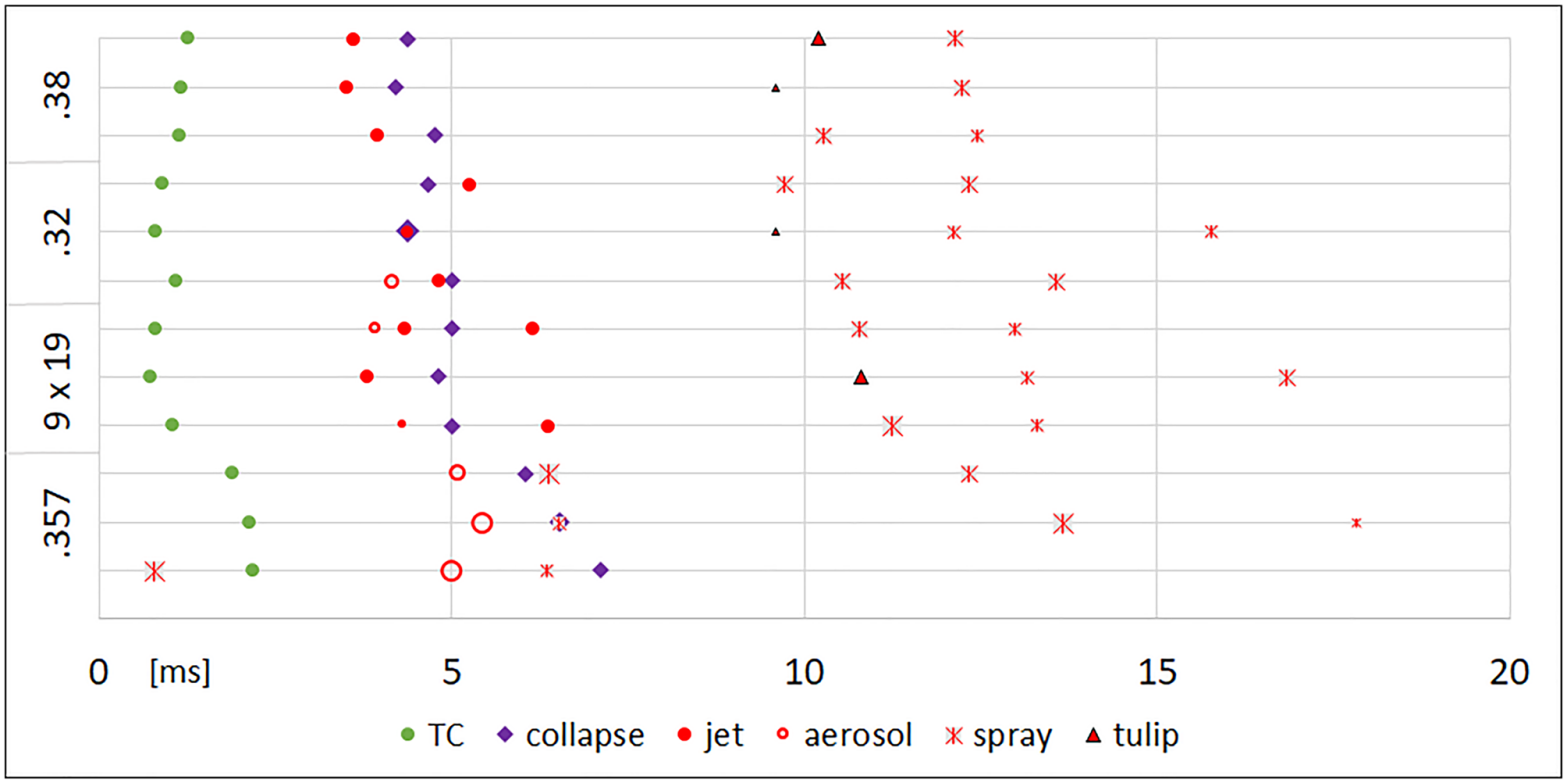
Timeline of the phases of fluid ejection in shots from 2 cm distance. The markers are roughly scaled to underline the large variation of observations

.357 Magnum ammunition was shot using a Smith & Wesson revolver with a four inch barrel. Linear jets of liquid were observed in shots from 10 and 5 cm distance. Two shots from 3 cm distance showed a whiplash-like jet, which was followed by large spray. Another 3 cm shot caused aerosol-like escape of fluid, which transformed into spraying. This event was also characteristic for a 2 cm shot distance (Fig. 9). The variability of findings increased when the revolver was fired at 1 cm or at close contact. Large liquid amounts escaped from the target model, often preceded by a thick fog of paint aerosol.

### Velocity of back spattered fluid

More than 500 frames were selected from 102 video records to perform 284 velocity measurements of ejected paint. Whereas this was easy for the typical jet (Fig. 2A, Online Resource 2, Fig. 6), droplets required tracking by simultaneously watching the video to retrieve the same droplet in different frames. Loss of velocity was visible with linear jets. An extra long video of a .38 special shot documented, for example, an initial jet velocity of about 20 m/s which decreased to 1 m/s within a distance of half a meter. Generally, the highest velocity of backspatter was recorded in the first phase of ejection. Further escaping liquid material was slower. However, near contact shots and close contact shots partly produced an aerosol, in which paint was sprayed without resolution of droplets. That is why it was only possible to measure the progress of the quickly expanding cloud. As a consequence, these velocities might be underestimated as a result of limited resolution and visibility. This issue should be kept in mind when the results are compared.

The velocity of jets produced by shots from 5 cm and greater distances varied in a similar range for all calibers (Table 1), independent of the shot range. Spraying with higher velocities was observed from 3 cm to contact for 9 mm Luger and .357 Magnum and from 2 cm to contact using .32 auto and .38 specials. Overall, there was a large spectrum of results for all shot ranges and a noticeable variation even when using the same firearm and ammunition.

**Table 1:** Velocity of back spattered liquid for different shot distances based on 284 measurements

Shot range	≥ 5 cm	3 cm	2 cm	1 cm	contact
Calibre	Velocity range [m/s]				
.38 special	5.7 – 26.9	6.7 – 10.4	17.4 – 31.5	26.8 – 45.4	39.4 – 79.9
.32 auto	6.0 – 45.2	9.2 – 18.0	14.5 – 36.3	33.0 – 156.8	118.7 – 305.3
9 mm Luger	7.4 – 25.5	31.4 – 46.2	18.4 – 191.8	84.1 – 153.3	107.1 – 193.8
.357 Magnum	8.7 – 44.3	27.2 – 66.2	27.6 – 38.6	35.1 – 81.4	46.4 – 329.9

## Discussion

In general a bullet is decelerated whilst passing through a soft target medium. The kinetic energy lost is transferred to the target medium. In transparent gelatin models, it is possible to observe this process using high-speed cameras [12]. Gelatin is radially displaced forming a temporary cavity (TC), which collapses a few milliseconds later. Due to the elastic property of gelatin, it goes on to form another, smaller TC, which then takes longer to collapse and so on (Fig. 3). Summarizing, the gelatin movement in all directions corresponds to a decreasing oscillation until all deposited energy is consumed. In order to simulate contact shots in gelatin, the blocks had to be covered on the entry side to force the muzzle gases to enter into the block. This was achieved using a "reference cube" [10]. This 12-cm long head surrogate is covered by an absorbent kitchen wipe beneath which a flat reservoir of viscous acrylic paint is fixed. High-speed video (HSV) of shots to reference cubes showed not only the bullet-"tissue" interaction with the expanding and collapsing TC, but also what happened to the fluid sealed in the paint pad.

Astonishingly, at the moment when the bullet penetrated the model and perforated the paint pad, retrograde ejection of paint was never observed. In distant shots, only a short crown-like protrusion with back spattering surface material was observed for approximatively 0.2 ms beginning with the bullet penetration (Fig. 1). This result corresponds to the findings of Radford et al. [15] who shot live anaesthetized pigs using 9 mm Luger FMJ. Following their definition, this phenomenon might be interpreted as tail splashing (crushed material back streaming over the projectile). Black et al. described larger tail splashing caused by the penetration of the projectile, when they shot at bare gelatin blocks [16] (Online Resource 5). This confirms the importance of the tight cover used in the reference cube model [10]. Further, Radford et al. described a ballooning of the pig skin for 0.7 ms [15] which exactly matches the time of the ballooning of the front cover in the reference cube model.

All shots (n = 102) caused a retrograde ejection of liquid, but at a different point in time. The results indicate a marked difference between shots from distance (≥ 5 cm, n = 51) and (near) contact shots. This difference concerns the moment as well as the type and the velocity of liquid ejection.

Distant shots, independent of the caliber, provoked a linear jet, which started in the late TC's collapse phase or after its collapse. The deviation of the jet from straight backward (0°) was moderate and stochastically distributed. The backward travelling end of the jet was often curved like a tongue (Fig. 5, 6A, 6B). Once the jet had started, it continued in a band-like stream, sometimes exhibiting a slow twist (Online Resource 3). Finally the jet got thinner and broke off after about 60 to 100 ms, when the visible movement of the gelatin cube had stopped. In some shots, a bulb- or tulip-like widening was observed in the chronological context of the second TC's collapse. The velocity of the jet varied widely (6 – 45 m/s) and is not related to ammunition or shooting distance. Comiskey et al. published similar data derived from videos for blood pattern analysis using bare and covered sponge models [17, 18], although the image quality of their videos was inferior. The analysis of high-speed films of distant shots (9 mm Luger) to living calves resulted in initial blood drop velocities of 13 to 61 m/s [19]. In the Handbook of forensic medicine, Karger gives the order of 10 m/s [20]. Lazarjan et al. measured 21 to 37 m/s for the ejection of brain material after distant shots to slaughtered ovine and bovine heads [21].

In an article on backspatter published in 1931, Weimann mentioned the observation of hunters concerning a light reflex when the game was hit by the bullet [22]. It was interpreted as a blood stream from the entry wound. Considering the results obtained using handguns, it might be possible that a jet of blood caused by hunting ammunition could be perceived by the eyes.

When the muzzle to target distance decreased to 3 cm and less, the character of liquid ejection changed significantly. Aerosol-like substance escape as well as cone-like spray or spatter were observed. Mainly, the release of liquid was not continuous and occurred in several phases with higher velocities (up to 330 m/s). In contrast to distant shots, the result depended on ammunition and distance. At close range, it was obvious that muzzle gases were blown into the target model. The expansion of the TC was larger [14] and its collapse took longer. In contrast to distant shots, where a rhythmic undulation of the TC was observed, close range shots showed irregular movement after the first collapse of the TC. The hypothesis that muzzle gas might be trapped inside the gelatin, preventing a complete collapse of the TC, [14] was vividly confirmed by one or several gaseous eruptions, which followed the first collapse (Fig. 7). The results reflected the increasing influence of muzzle gas [14], which is determined by the cartridge, the barrel length [23, 24] and the gap between muzzle and target. The variable of the barrel length was eliminated by using only four inch barreled firearms in accordance with previously published articles [e.g. 13]. The influence of energy transfer was reduced by choosing exclusively non-deforming full metal jacketed bullets.

The study confirmed the results published by Wagner [25] who shot anaesthetized rabbits using a 7.65 mm Browning (.32 auto) from various distances up to contact. He reported a significant decrease of backspatter when the distance was 2.5 cm or more. The knowledge of muzzle gas effects that depend on cartridge load and distance in close range shots was already documented in the handbook of Hofmann 1881 [26]. Hofmann also mentioned experimental close range and contact shots to cadavers, which provoked backspatter of powder and tissue debris on the shooter's hand. Since then many forensic pathologists have investigated back spattered material on the hands, on the weapon, and inside the barrel after contact shots [e.g. 22, 27–29]. Experimental research used either biological targets (living rabbits [25], living calves [19], living pig and pig heads [15], human cadavers [30], ovine and bovine heads [21]) or blood soaked sponges [5, 31–33]. All these approaches have particular advantages, but the common disadvantage was that the targets are not transparent and are dissimilar to human heads. The investigation of staining inside firearm barrels initiated using non-transparent silicone covered hemispheres [34], boxes [24] or polyethylene bottles [35]. With introduction of the "reference cube" with 12 cm edge lengths and a weight of 1.7 kg [10], a cheap transparent gelatin target model was available for reproducible ballistic experiments without ethical issues. Even though the "reference cube" is dissimilar from a vital human head, this surrogate has allowed the study of staining inside firearm barrels [13] as well as wound ballistic effects [14, 36] and their influence on "backspatter".

## Conclusion

Backspatter is a complex phenomenon. The crushing effect and the energy transfer of the bullet leading to the temporary cavity are essential mechanisms working at any shot distance. In close range and contact shots however, the influence of muzzle gases increases with decreasing shot range. Muzzle gases change the timing, the characteristic and the velocity of back spattered material. The complex and yet not predictable interaction of muzzle gases and liquefied tissue material might explain the heterogeneity of observations in forensic practice.

## Key points

1. The "reference cube" containing a paint pad beneath a cover is a suitable target model to study "backspatter" because its transparency allows tracking of muzzle gases as well as the propagation of fluid using high-speed video.
2. Short tail splashing of superficial material was observed in all distant shots as an immediate consequence of the bullet's penetration.
3. In distant shots (≥ 5 cm), the ejection of fluid started in connection with the collapse of the temporary cavity as a linear jet with moderate velocity (6 – 45 m/s).
4. Close range and contact shots provoked earlier and faster (up to 330 m/s) ejection of fluid against the line of fire, depending on the ammunition and the gap between muzzle and target. Typically, the fluid was sprayed backwards in a cone shape or escaped as quickly expanding aerosol. Muzzle gases disturbed the collapse and the further oscillation of the temporary cavity. The interaction of muzzle gases and fluid was obvious.
5. Although all shots were fired following strictly standardized conditions, it was possible to demonstrate individual variations for shots fired with the same firearm and ammunition.

## Electronic supplementary material

Below is the link to the electronic supplementary material.

Online Resource 1 9 mm Luger full metal jacketed bullet penetrating into the target model (covered by a pink kitchen wipe) and causing tail splashing. 25 μs between eachframe

Online Resource 2 Inverted picture of another .32 auto full metal jacketed bullet penetrating into the covered target model and causing tail splashing. 25µs between each frame.

Online Resource 3 Examples of twisted, descending jets:
A: .38 special, 32.0ms, 5.6m/s
B: 9 mm Luger, 17.7ms, maximum velocity 31.4m/s (measured at 4.5ms) decreased to 12.5m/s (picture).

Online Resource 4 .32 auto fired from 1cm distance. First (0.5ms) a fine aerosol, followed by fast spray (1ms) and abundant spatter at 4.6ms.

Online Resource 5 .357 Magnum full metal jacketed bullet penetrating a bare gelatin block causing tail splashing.
