# Supplementary figures and images for: Study of backspatter using high-speed video of experimental gunshots
Source: Forensic Sci Med Pathol. 2020 Dec 14;17(1):36–46. doi: 10.1007/s12024-020-00326-0 (PMC7889577; doi:10.1007/s12024-020-00326-0)

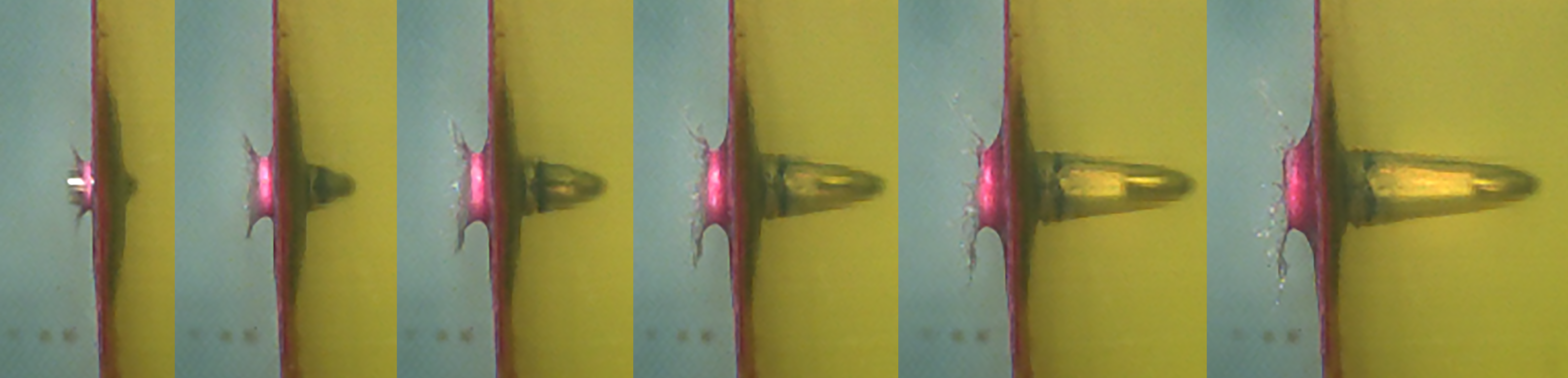

Supplement: Supplementary file 1 — 9 mm Luger full metal jacketed bullet penetrating into the target model (covered by a pink kitchen wipe) and causing tail splashing. 25 μs between eachframe [file 12024_2020_326_MOESM1_ESM.tif]

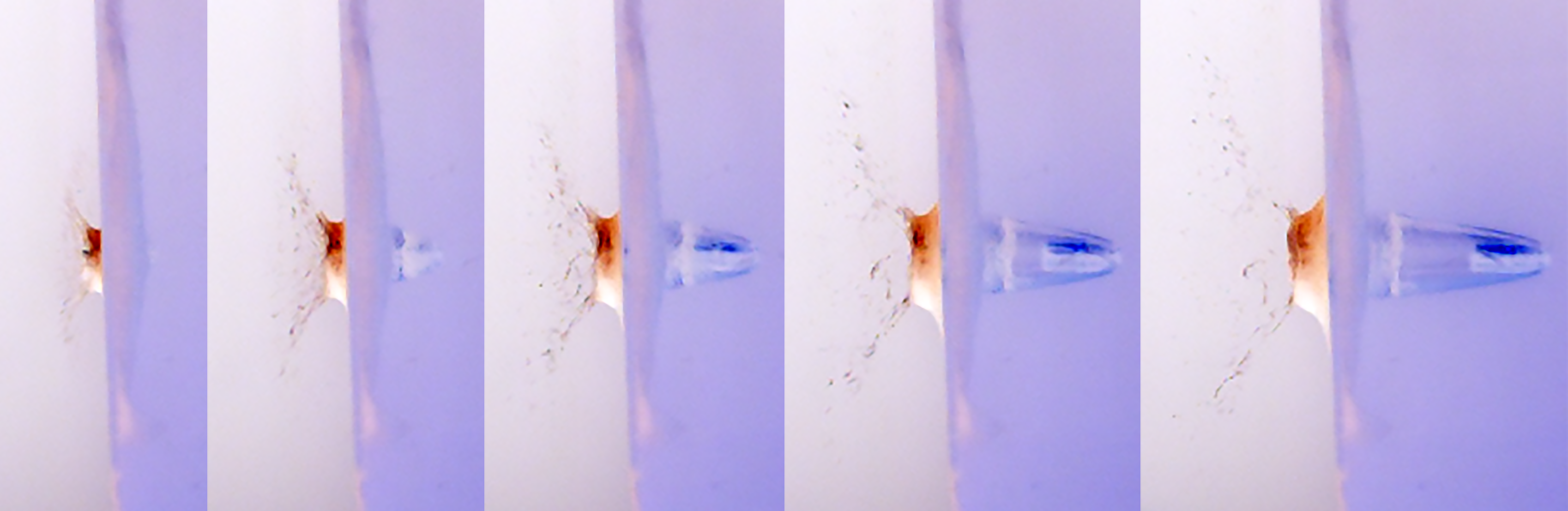

Supplement: Supplementary file 2 — Inverted picture of another .32 auto full metal jacketed bullet penetrating into the covered target model and causing tail splashing. 25µs between each frame. [file 12024_2020_326_MOESM2_ESM.tif]

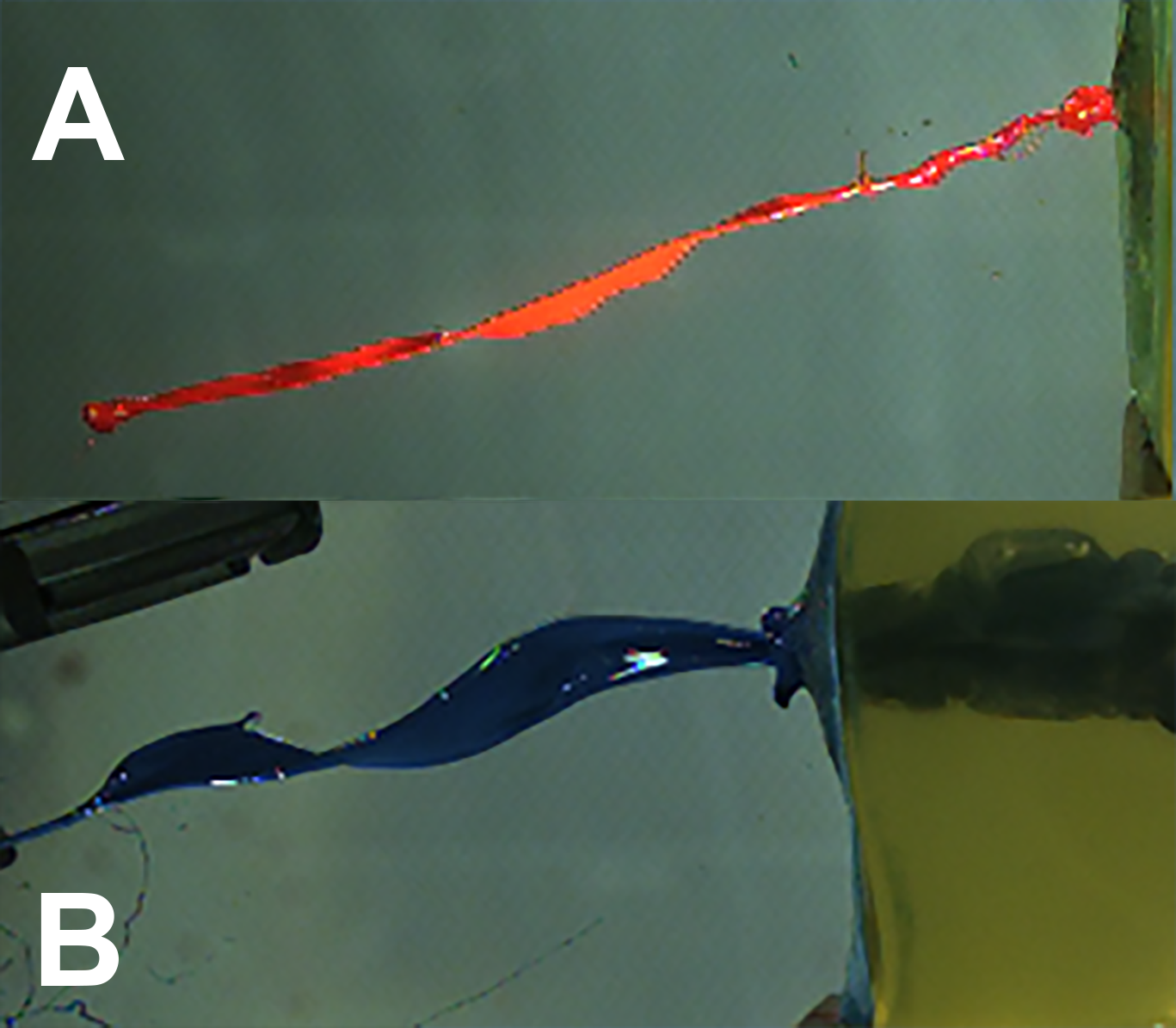

Supplement: Supplementary file 3 — Examples of twisted, descending jets: A: .38 special, 32.0ms, 5.6m/s B: 9 mm Luger, 17.7ms, maximum velocity 31.4m/s (measured at 4.5ms) decreased to 12.5m/s (picture). [file 12024_2020_326_MOESM3_ESM.tif]

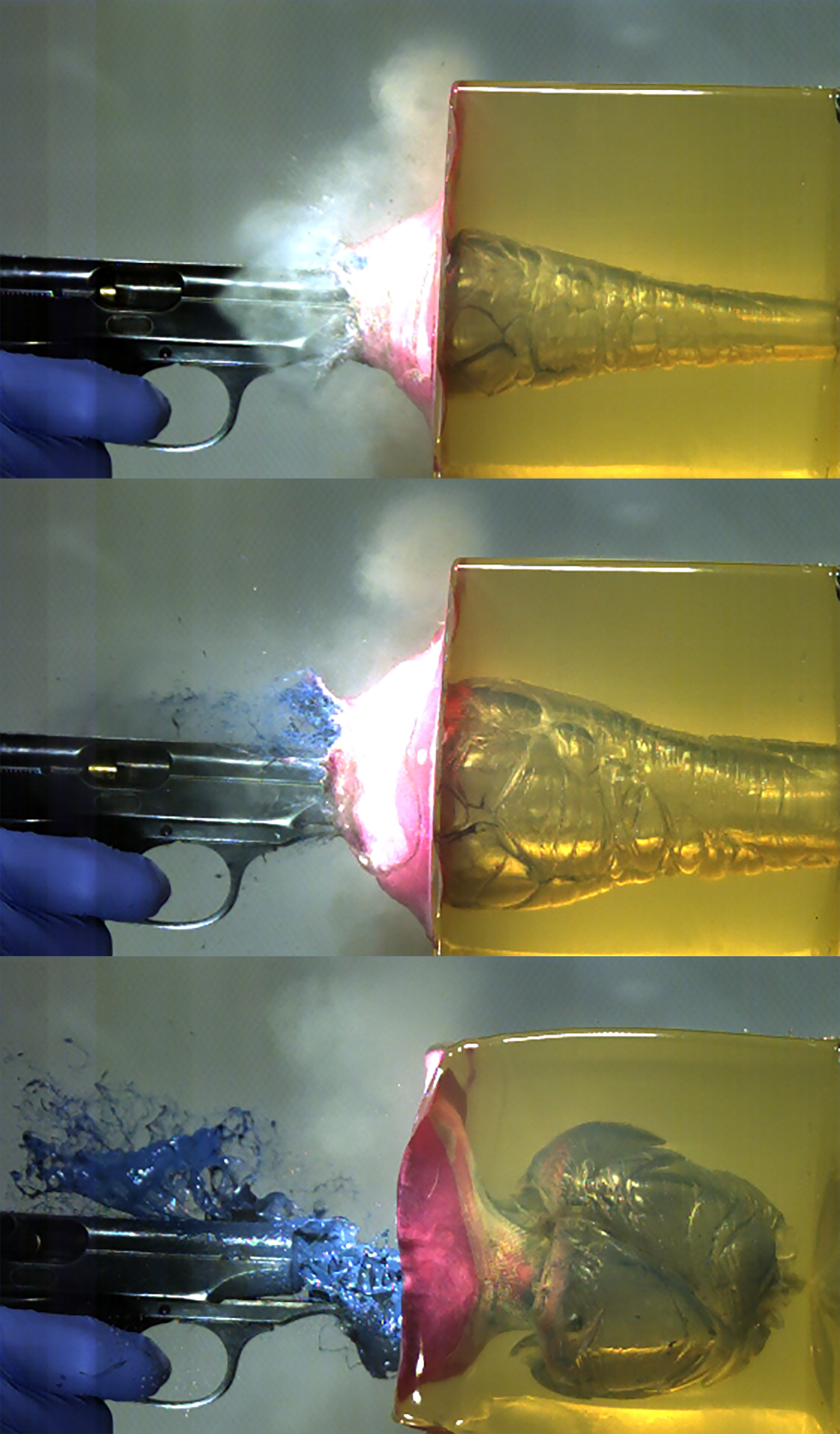

Supplement: Supplementary file 4 — .32 auto fired from 1cm distance. First (0.5ms) a fine aerosol, followed by fast spray (1ms) and abundant spatter at 4.6ms. [file 12024_2020_326_MOESM4_ESM.tif]

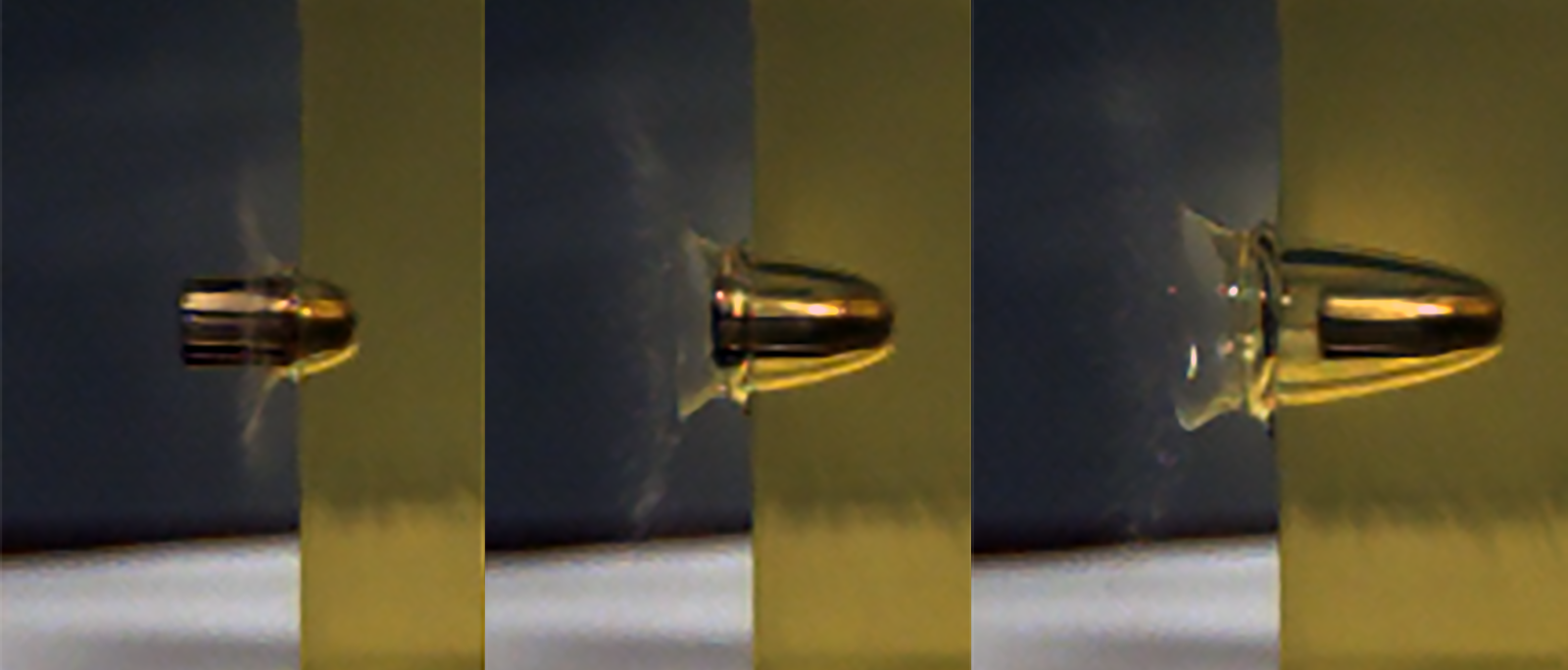

Supplement: Supplementary file 5 — .357 Magnum full metal jacketed bullet penetrating a bare gelatin block causing tail splashing. [file 12024_2020_326_MOESM5_ESM.tif]
